# Barriers to Colorectal Cancer Screening and Surveillance in Homeless Patients: A Case Report and Policy Recommendations

**DOI:** 10.1097/AS9.0000000000000183

**Published:** 2022-08-02

**Authors:** Hope E.M. Schwartz, Mary Kathryn Abel, Joseph A. Lin, Hannah C. Decker, Margot B. Kushel, Elizabeth C. Wick

**Affiliations:** From the * University of California, San Francisco School of Medicine, San Francisco, CA; † Department of Surgery, University of California, San Francisco, San Francisco, CA; ‡ Department of Medicine, University of California San Francisco, San Francisco, CA.

**Keywords:** colonoscopy, colorectal cancer, homeless, policy, screening, surveillance

## Abstract

Patients experiencing homelessness face a high burden of chronic disease, including colorectal cancer. Access to colonoscopy is limited by many structural barriers in this population. In an exemplar case, we describe the barriers encountered by a homeless patient with a history of colorectal cancer who was lost to follow up and presented 11 years later with a new primary colon cancer. We provide policy solutions to increase the use of primary and secondary screening, including essential private bathroom access for colonoscopy preparation in patients who had a positive screening or require surveillance after diagnosis and treatment. We believe that increasing early detection and treatment may be cost-effective and could reduce disparities in morbidity and mortality in homeless patients.

## Abstract 2

Mini-abstract: Patients experiencing homelessness face significant barriers to screening and treatment for colorectal cancer, leading to worse outcomes. In this perspective, we use an exemplar patient case to highlight potential policy solutions for reducing this health care disparity by increasing access to early detection and treatment in this population.

Despite the efficacy of screening and surveillance colonoscopy for primary and secondary prevention, rates of colorectal cancer remain high in vulnerable populations.^1^ The US Preventive Services Task Force recommends screening colonoscopy for adults over 50 years old, with more frequent screening after a diagnosis of colorectal cancer; however, guidelines are difficult to follow even for well-resourced patients.^2^

Barriers to colorectal cancer screening in vulnerable populations include less access to and utilization of primary care, lower rates of provider recommendation, limited health literacy, and distrust in the healthcare system.^3^ Patients experiencing homelessness face additional structural barriers, including lack of private space to complete bowel preparation and competing priorities, which may contribute to the lower rates of colorectal cancer screening in homeless populations.^4^ Increasing access to colonoscopy for both primary and secondary prevention is important to address inequity in this population. Drawing on the clinical course of a homeless patient with colorectal cancer, who experienced suboptimal secondary prevention and a new cancer, we highlight key structural barriers and propose policy solutions.

## CASE

Our patient is a 64-year-old chronically unhoused man. In 2006, he was diagnosed with synchronous bulky mid-rectal and sigmoid adenocarcinomas and underwent radiation, surgical resection (low anterior resection with diverting loop ileostomy), and partial adjuvant chemotherapy (discontinued because of intolerance) followed by ileostomy reversal. He underwent a follow-up surveillance colonoscopy in 2008, which was normal except one small adenomatous polyp which was removed. He was lost to follow up in 2009 and did not receive recommended surveillance colonoscopies for secondary prevention. In March 2020, he was admitted for COVID-19 and was incidentally found to have severe microcytic anemia. He was discharged to a hotel to isolate, which facilitated bowel preparation for colonoscopy, and a large mass was found in his transverse colon. Biopsy revealed invasive adenocarcinoma. Given his prior colon cancers, this was concerning for Lynch syndrome, but the patient deferred genetic counseling or testing. Due to his COVID infection, the patient was discharged to city-funded housing, and staging computed tomography (CT) scans of the chest, abdomen and pelvis were coordinated. We recommended resecting the cancer and his entire remaining colon, leaving a permanent ostomy. However, out of concern for ostomy care without stable housing, he elected for a segmental colectomy. He was preadmitted the day before surgery and underwent a mechanical bowel preparation with oral antibiotics in the hospital. At the time of surgery, the hepatic flexure tumor was large and abutting the duodenum and head of the pancreas but was able to be freed with negative margins and an extended right colectomy with primary anastomosis was done. The final pathology demonstrated a T4N0 (0/27 lymph nodes), moderately differentiated adenocarcinoma, with mucinous features and perforation. The tumor was tested and showed high microsatellite instability, increasing suspicion for Lynch syndrome, but again the patient deferred genetic testing.

After surgery, since he had cleared his COVID infection and could no longer return to the pandemic-specific housing, we discharged him to homelessness. He was unable to return for timely follow-up, and thus missed the window for adjuvant chemotherapy. We instead planned an intensive prospective surveillance program given the concern for Lynch syndrome, including imaging every 3–6 months for 5 years and colonoscopy every 1–2 years. His housing situation has stabilized, and his follow-up to date has included 3 CT scans with no evidence of metastatic or recurrent cancer.

## DISCUSSION

Screening and surveillance colonoscopies are essential diagnostic and treatment approaches for colorectal cancer, but access is inequitable. This case highlights structural barriers to guideline adherence for colorectal cancer surveillance in a homeless patient. Temporary noncongregate shelter for COVID isolation facilitated our patient’s ability to receive a colonoscopy, leading to his second cancer diagnosis. The optimal oncologic management would have resulted in a permanent ostomy, which was not practical given his unstable housing status. Delayed follow-up after surgery, in part related to difficulty reaching the patient after discharge without stable shelter, meant that he did not have the option to complete adjuvant chemotherapy and improve his survival. Furthermore, with the operative approach selected, he still has considerable colon remaining and thus is at heightened risk for another colorectal cancer occurrence. To prevent this, he has been recommended intensive colonoscopic surveillance, which is likely unfeasible.

Colorectal cancer is preventable and treatable with early detection and guideline-concordant follow-up. Increasing access to colonoscopy is critical given the aging homeless population in the United States. Colorectal cancer is on the rise even in younger patients, and homeless patients are at risk of accelerated aging due to environmental and psychological stressors.^5^ Together, these factors highlight the importance of screening homeless patients at or even below current age guidelines.

**FIGURE 1.**
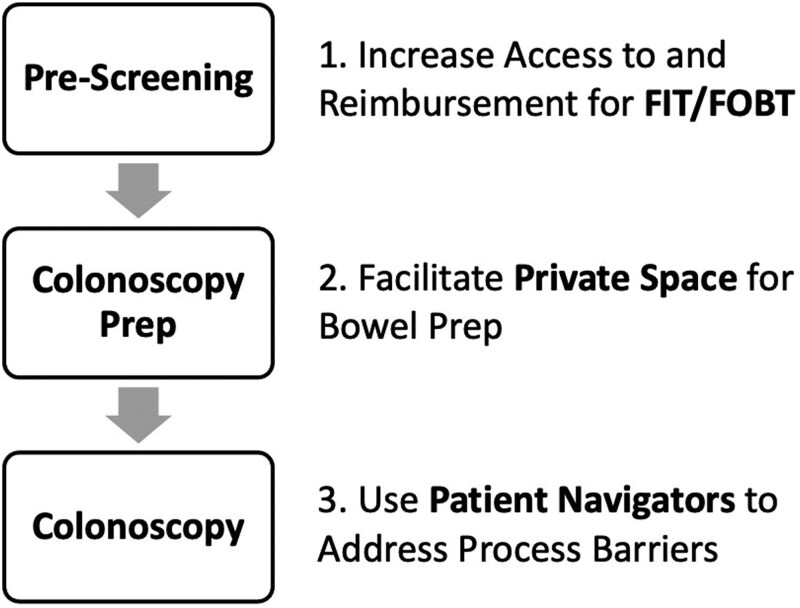
Proposed policy solutions for increasing colorectal cancer screening in homeless patients.

Our patient did not receive follow-up surveillance after his first colorectal cancer diagnosis in 2006, likely contributing to the advanced presentation of his subsequent colorectal cancer in 2020. Now, his health is highly dependent on accessing regular colonoscopy and imaging. This case raises a vital policy question: how can the healthcare system improve access to colonoscopy in homeless populations? Colorectal cancer screening is only one example of gaps in care for homeless patients, and improving overall access to healthcare maintenance and diagnostic services would address many of the barriers specific to colorectal cancer screening. Solutions require engagement with governmental and community groups at the local, state, and federal levels, as well as collaboration with provider and insurer networks. As direct witnesses to the health impact of these barriers, surgeons are in a unique position to bring the patient care perspective to the multistakeholder discussion. Based on this case and prior literature, we suggest three specific policy goals to improve colorectal cancer screening in this population (Fig. 1).

### Facilitate Fecal Immunochemical Test and Fecal Occult Blood Test Testing Use for First-Line Screening

The fecal immunochemical test (FIT) and fecal occult blood test (FOBT) are yearly tests that are routinely used for primary prevention in safety net systems across the United States. They require a single stool sample and can be completed in shared or public bathrooms. FIT and FOBT testing provides an affordable and accessible screening option for homeless patients and limits the number of colonoscopies needed because only positive tests require colonoscopy follow-up. While not appropriate for secondary prevention, as in the case of our patient, increased use of alternative screening modalities reduce primary cancers in this population.

Key barriers to optimal use of FIT and FOBT in homeless patients include patient nonadherence and providers not recommending screening when follow-up colonoscopy is virtually impossible. Interventions aimed at increasing adherence to FIT and FOBT often involve mailing reminders and testing kits to patients’ home addresses, which is not feasible for in this population.^6^ However, interventions have shown promise in homeless patients. Hardin et al^7^ (2020) studied a three-pronged intervention including small financial incentives, patient navigators, and patient reminders at a Federally Qualified Health Center that increased FIT return rate from 22% to 49%. While yearly FIT or FOBT tests are covered by Medicare starting at age 50 (in line with USPSTF recommendations), Medicaid coverage varies by state. Given that younger homeless patients are likely to be insured by Medicaid, standardizing Medicaid coverage may also increase screening for homeless patients.

### Increase Access to Private Bathrooms for Colonoscopy Bowel Preparation

While FIT and FOBT may be effective screening approaches in this population, patients with positive results require follow-up colonoscopy. Providers may be more hesitant to initiate screening given the complexity of arranging colonoscopy follow-up on a positive test.^3^ In a survey of homeless patients within the recommended screening age range, only 37% reported discussing colonoscopy with their providers, compared to 75% of housed patients.^8^ Homeless patients are more likely to decline colorectal cancer screening, with discomfort and lack of access to private bathrooms as commonly cited barriers.^4^ In our patient’s follow-up care, access to regular colonoscopy was critical after his initial diagnosis and continues to be a key aspect of his ongoing management. Importantly, access to private and clean bathrooms is relevant not only for colonoscopy preperation but also for many other healthcare needs including postsurgical wound care, as well as tube and drain management.

Mechanical bowel preparation for colonoscopy involves taking oral laxatives over a 24-hour period and requires frequent access to a toilet. Poor bowel preparation reduces the diagnostic sensitivity of colonoscopy or may even require repeating the procedure.^4^ In our health system, providers are occasionally able to preadmit patients for bowel preparation in cases of strong family or personal history. However, this process is logistically difficult, cost-prohibitive, and often unsuccessful. Medical respite programs are a potential alternative, providing short-term housing for patients who are homeless and undergoing medical treatment. However, access to respite has not improved colorectal cancer outcomes for homeless patients.^9^ Most respite centers have shared bathrooms and, in our experience, have refused patients for bowel preparation because of limited bathroom capacity. Increased access to comfortable, private space for bowel preparation will require more substantial intervention including colonoscopy-specific respite programs or hotel vouchers. Given the high economic burden of colorectal cancer, interventions aimed at providing optimal spaces for colonoscopy preparation for patients with positive FIT or FOBT and other high-risk patients may prove cost effective by facilitating early detection and treatment.

### Incorporate Patient Navigators in Screening and Surveillance

Our patient was discharged with a complex plan for follow-up care that will require regular imaging and colonoscopy. Screening and surveillance for colorectal cancer are difficult for any patient to coordinate, and homelessness may further impair patient adherence to recommendations. Interventions aimed at providing navigational support for homeless cancer patients are important to improving utilization, especially in patients such as ours who require multistep follow-up care. Patient navigators, who assist patients with scheduling and accompany patients during and after appointments, have been shown to increase screening colonoscopy in low-income groups.^10^ In addition to care coordination, the medicolegal need for postsedation escort on discharge is an additional barrier. Programs using trained Community Health Workers add value by providing culturally competent support for patients to address fear and discomfort, as well as reducing the logistical burden of arranging appointments and finding escorts.^10^ Increasing access to these services would likely improve adherence to evidence based screening and treatment beyond colorectal cancer screening.

## CONCLUSION

In addition to general barriers to accessing healthcare for homeless patients, specific structural barriers to colonoscopy increase the risk of colorectal cancer and prevent postdiagnosis surveillance in this population. Research on the patient perspective in this process is limited and will be important to prioritizing policy implementation. Our patient’s loss to follow up for colorectal cancer surveillance likely led to increased morbidity at re-presentation and necessitated a complex care plan. We recommend specific policy action to address key barriers: facilitate FIT and FOBT use, restructure medical respite or provide hotel vouchers for private bathroom bowel preparation for high-risk patients, and fund patient navigator programs. Ultimately, comprehensive interventions aimed at creating stable permanent housing and wraparound healthcare services is key to addressing the multiple factors underlying this and other health care disparities for homeless patients. As front-line providers, surgeons have a unique perspective on the real health impact of these structural inequities and are well positioned to serve as advisors and advocates in government, nonprofit, and private sector organizations addressing homelessness in the United States.
